# Psychological characteristics and associations between living kidney transplantation recipients and biologically related or unrelated donors

**DOI:** 10.1186/s12882-020-02017-y

**Published:** 2020-08-20

**Authors:** Yujin Lee, Hyewon Park, Hee-Jung Jee, Heon-Jeong Lee, Jun Gyo Gwon, Hyeonjin Min, Cheol Woong Jung, Myung-Gyu Kim, Chul-Hyun Cho

**Affiliations:** 1 Department of Psychiatry, Seoul Metropolitan Eunpyeong Hospital, Seoul, South Korea; 2 Department of Psychiatry, Samsung Biomedical Research Institute, Seoul, South Korea; 3 Department of Biostatistics, Korea University College of Medicine, Seoul, South Korea; 4 Department of Psychiatry, Korea University College of Medicine, Seoul, South Korea; 5 Department of Surgery, Korea University College of Medicine, Seoul, South Korea; 6 Division of Nephrology, Department of Internal Medicine, Korea University College of Medicine, Seoul, South Korea; 7 Department of Psychiatry, Chungnam National University Sejong Hospital, Sejong, South Korea; 8 Department of Psychiatry, College of Medicine, Chungnam National University, 82 Munhwa-ro, Jung-gu, Daejeon, 35015 South Korea

**Keywords:** Kidney transplantation, Recipient, Donor, Psychology, Depression, Anxiety, Psychological association

## Abstract

**Background:**

Although recipients and donors in living kidney transplantation experience psychological distress—including depression and anxiety—during the pre-operative period, very few studies have evaluated the related psychological reactions. This study aimed to determine the characteristics and correlations of the mood states and personality of recipients and donors (genetically related and unrelated) of living kidney transplantations.

**Methods:**

A total of 66 pairs of living donors and recipients were enrolled from April 2008 to June 2019 in this study, of whom 53 eligible pairs of living donors and recipients were included in the retrospective analysis of their psychological assessments in the pre-transplantation states. While participants’ personality patterns were assessed using the Minnesota Multiphasic Personality Inventory-2 (MMPI-2), mood states were evaluated via both the State-Trait Anxiety Inventory (STAI) and The Center for Epidemiologic Studies Depression Scale (CES-D). Statistical analysis was performed using paired *t*-tests and Spearman’s correlation analyses.

**Results:**

The recipient group showed significantly higher scores for Hypochondriasis (*t* = − 4.49, *p* = .0001), Depression (*t* = − 3.36, *p* = .0015), and Hysteria (*t* = − 3.30, *p* = .0018) of MMPI-2 and CES-D (*t* = − 3.93, *p* = .0003) than the donor group. The biologically unrelated recipient group reported higher scores of Hypochondriasis (*t* = − 3.37, *p* = .003) and Depression (*t* = − 2.86, *p* = 0.0098) than the unrelated donor group. Higher scores for Hypochondriasis (*t* = − 3.00, *p* = 0.0054) and CES-D (*t* = − 3.53, *p* = .0014) were found in the related recipient group. A positive association was found for Hypomania (*r* = .40, *p* = .003) of MMPI-2, STAI-S (*r* = .36, *p* = .009), and CES-D (*r* = .36, *p* = .008) between the recipient and donor groups.

**Conclusions:**

Recipients suffered from a higher level of depression and somatic concerns than donors before living kidney transplantation. Psychological problems like depression and anxiety can occur in both living kidney transplantation donors and recipients. This study suggests that clinicians must pay attention to mood states not only in recipients but also in donors because of emotional contagion.

## Background

Kidney transplantation is the treatment of choice for end-stage renal disease (ESRD) as it had proven effective in ensuring survival and enhancing quality of life compared to maintenance dialysis [1]. Kidney transplantation may be divided into those with living or deceased donors. More than 27,000 living-donor kidney transplantations are performed each year across developed and developing countries [2]. According to the International Registry in Organ Donation and Transplantation (IRODaT) in 2017, the number of deceased organ donors was 9.95 per million people. In contrast, the number of living donors is 44.28 per million people in South Korea. Of the 25,101 total kidney transplantation from February 2002 to June 2019, 15,541 (61.9%) were living kidney transplants [3], indicating that living kidney transplantation is more common than deceased kidney transplantation in South Korea.

Studies of various aspects of only one group of donors or recipients in living kidney transplantation have been conducted. First, regarding recipients, it is known that mortality in patients with depression after kidney transplantation is higher than in patients without depression [4, 5]. Depression is associated with a two-fold increase in the risk of graft failure and death [6]. Although kidney transplantation is known to cause lower psychological stress than hemodialysis [7, 8], one-fifth of transplant recipients are still at high risk of clinically significant depression [9]. This may be due to both ESRD and feelings of guilt in recipients with respect to donors [10–12].

Studies of donors’ psychological difficulties report that while the majority of donors experience neither depression (77–95%) nor anxiety (86–94%), 39% of the donors described the overall experience as being at least somewhat stressful [13]. Another study, however, suggests that poor recipient outcomes may result in depression in donors, feelings of waste and guilt, and even conflicts in donor–recipient relationships [14].

Donor–recipient relationships might be collaborative interactions in which an individual donates an organ to the other [15]. Transplantation within a family requires the donor and recipient to psychological support each other throughout the transplantation process. In the clinical field, clinicians should consider the mood and anxiety states, personality, and coping skills of donor and recipient individually, as well as their interaction and association with each other. In a systematic review of clinical practice guidelines [16], the relationship between the potential donor and the recipient should be evaluated to address the issues of imbalance of psychological dynamic, conflict, and expectations. Prior to transplantation, psychosocial assessment is essential, and the donor’s psychological status and history including depression, anxiety, and medication dependency should be evaluated. Furthermore, clinicians should determine whether they have adequate social support and coping mechanisms [17, 18].

However, the psychological characteristics and associations between donors and recipients in living kidney transplantation have received less attention. A recent study showed the relational experiences within living donor kidney transplantation dyads through in-depth interviews, showing that for each donor-recipient dyad, the transplantation process reflects the unique broader social and interpersonal context within which the dyad evolves, suggesting that the decision to give or accept a kidney reflects the social role one adheres to with respect to the other person in everyday life [19]. Another study reported that recipients felt guilty and indebted to the donor and did not want to disappoint the donor in case the kidney failed [20]. However, there has been no study of mood or personality associations between donors and recipients of living kidney transplantation.

Our center examined MMPI, STAI-T, STAI-S, and CES-D scores obtained during the psychological screening of all the donors and recipients at the pre-transplantation state. During the evaluation process, we have faced some critical questions. First, were there any differences in mood symptoms, such as depression or anxiety, between the recipient and the donor group in living kidney transplantation? The recipient group is expected psychological difficulties such as depression and anxiety due to chronic diseases. However, donors could also suffer from the stress of having to undergo surgery and financial challenges due to hospitalization. Therefore, we compared the scores on the MMPI, which can identify personality, and the degree of depression (CES-D) and anxiety (STAI) in these two groups. Thus, under the hypothesis that the depression and anxiety levels of the recipient group are higher than in the donor group, we compared the degree of depression (CES-D) and anxiety (STAI) with the personality characteristics as identified by the MMPI.

The second question was whether the psychological states of the donor and the recipient were correlated. In South Korea, 95.7% of living donor kidney transplantation occurs in familial relationships [3]; therefore, we assumed that the scores of anxiety and depression using CES-D and STAI of the recipient would be related to the degree of anxiety and depression of the donor. Psychological difficulties such as depression and anxiety are associated with a worse prognosis in kidney transplantation. Therefore, if there are associations between the donor’s and the recipient’s moods, this would have clinical importance when considering the interaction between the donor and the recipient’s mood, rather than considering the recipient’s and donor’s moods separately. Under the hypothesis that the mood states and personality characteristics of donors and recipients are linked, the associations of the two groups were examined.

The third question was whether there are differences in psychological characteristics such as personality or mood status depending on whether the donor is biologically related or unrelated to the recipient. According to the New Ethical, Legal, and Psychosocial Aspects of Organ Transplantation (ELPAT) classification for living organ donation [21], living organ donation in South Korea is mainly distinguished as “donation to genetically and emotionally related recipient (e.g., to one’s child, parent, or sibling)” and “donation to genetically unrelated but emotionally related recipient (e.g., to one’s spouse, friend, or acquaintance).” Of the 1260 living kidney transplantation cases conducted in 2018 in South Korea, 431 (34.21%) cases were transplanted by a spouse. Therefore, genetically unrelated but emotionally related living kidney transplantation accounts for a significant proportion of living kidney transplantation. HLA mismatching is more prevalent in biologically unrelated kidney transplantations than biologically related kidney transplantations. However, recent studies have shown no significant difference in survival rates between biologically related and biologically unrelated living kidney transplantation [22–24]. It has been reported that spousal living kidney transplantation can strengthen marriage bonds, increase spouses’ fidelity to their roles as husbands and wives, and improve their relationship as a couple and with their children [25, 26]. On the other hand, it was assumed that biologically related donors and recipients might be related in temperament and personality, which would also affect coping skills because genetic factors influence temperament. Furthermore, recipients and donors are likely to have been exposed to similar environments, such as parenting, social-economic status, and family norms, which can lead to similar personalities or coping mechanisms. Therefore, we sought to examine how the association between the donor and recipient’s mood symptoms and personality characteristics differed depending on whether the recipient’s relationship with the donor was biologically related or unrelated.

This study aimed to investigate the characteristics and correlations of the mood (depression, anxiety) and personality of living kidney transplantation donors and recipients (genetically related and unrelated) to answer these questions.

## Methods

### Study population

Clinical data from adults aged 18 years or above at the time of transplantation who underwent living kidney transplantation at the Korea University Anam Hospital between April 2008 and June 2019 were included in the study. In South Korea, the pre-transplantation evaluation process to establish eligibility must include psychological assessment. All the candidates for living donor kidney transplantation were examined in the pre-transplantation psychological evaluation process at Korea University Anam Hospital. We analyzed the psychological assessment retrospectively.

Initially, 66 recipients and 66 paired kidney donors were examined. Clinical data from 26 foreigners who were unable to understand and complete the psychological questionnaire were excluded. Thus, we retrospectively reviewed the medical records of 106 adult donors and recipients who underwent living donor kidney transplantation at the Korea University Anam Hospital between April 2008 and June 2019, and all 53 pairs of living donors and recipients were included. While 31 pairs were biologically related, the remaining 22 were biologically unrelated but emotionally related.

The Institutional Review Board of the Korea University Anam Hospital (IRB No. 2019AN0380) approved this study. The principles described in the Declaration of Helsinki were followed during both clinical and research activities.

### Assessment

Several standardized questionnaires with known validity and reliability were employed to assess the severity of anxiety and depression symptoms, as well as the health information of transplant donors and recipients. All data were collected in face-to-face interviews by well-trained psychologists and then validated by expert psychiatrists.

### Assessment of socio-demographic information

Participants’ socio-demographic and general health information was collected. Specifically, the questionnaire was used to elicit the socio-demographic and clinical identification of patients, with the following data being recorded: name, age, gender, alcohol consumption, and smoking history (Table 1).

**Table 1:** Demographic variables (Mean± *SD* or *N* (%))

Variables	Recipients (*n* = 53)	Donors (*n* = 53)
Age (years)	46.98(11.27)	49.36(11.31)
Gender		
Male	34 (64.15%)	22 (41.51%)
Female	19 (35.85%)	31 (58.49%)
Alcohol history		
None	36 (67.92%)	
1 year	15 (28.3%)	
Currently in progress	2 (3.77%)	
Smoking history		
None	39 (73.58%)	
1 year	9 (16.98%)	
Currently in progress	5 (9.43%)	
Height	165.58(8.84)	
Weight	65.44(3.91)	
BMI	23.65(3.42)	

*BMI* body mass index

### Assessment of personality dimensions

The Minnesota Multiphasic Personality Inventory-2 (MMPI-2) is a well-standardized self-report measure designed to assess personality traits and psychopathology in an individual’s personality. Briefly, it consists of 567 statements that can be rated as “correct” or “incorrect.” Successively, statements are grouped into 10 clinical scales and 9 validity scales. This study is based on the results obtained from the following 10 clinical scales: Hypochondriasis (Hs), Depression (D), Hysteria (Hy), Psychopathic Deviate (Pd), Masculinity-Femininity (Mf), Paranoia (Pa), Psychasthenia (Pt), Schizophrenia (Sc), Hypomania (Ma), and Social Introversion (Si). Additionally, data from the following three validity scales are discussed: Lie (L), Infrequency (F), and Defensiveness (K). Rather than personality dimensions, the three validity scales assess either the individual’s pattern of responses or response bias. In contrast, the clinical scales assess a variety of clinical conditions (e.g., depression, anxiety, and psychopathic deviation) and are used to identify individuals with psychiatric symptoms.

In the present investigation, MMPI-2 raw scores were converted to *T*-scores to enable comparison with the normative group (standardized test) [27]. Given the *T*-scores of the normative group (mean = 50, *SD* = 10), a T-score between 50 and 65 is considered to be within the normal range. In contrast, a *T*-score ≥ 65 is interpreted as clinically significant. All the clinical scales of reliability and validity have been well-established [28]. Here, we used the Korean version of the MMPI-2, which has been highly validated [29].

### Assessment of anxiety and depression

The State-Trait Anxiety Inventory (STAI) is a 40-item self-report instrument assessing anxiety. Specifically, it consists of two subscales, one related to the anxiety state (STAI-S-20) and the other associated with the anxiety trait (STAI-T-20) [30]. Each item is scored on a 4-point Likert scale, and the overall score ranges from 20 to 80, with higher values indicating greater anxiety levels. We used the well-validated Korean version of both the STAI-S and STAI-T [31].

The Center for Epidemiologic Studies Depression Scale (CES-D) was applied to evaluate the symptoms of depression [32]. The 20-item CES-D assesses the frequency of depressive symptoms experienced in a week-long period on a 4-point scale (0 = *rarely*, 1 = *sometimes*, 2 = *moderately*, and 3 = *always*). The total score ranges from 0 (*no depressive symptoms*) to 60 (*severe depressive symptoms*) [33].

### Statistical analysis

To compare the psychological characteristics of renal transplant recipients and donors, we analyzed the psychological tests using paired *t*-tests. Also, an independent two-sample test was performed to evaluate the psychological differences between the biologically related and unrelated recipients and donors. Furthermore, Spearman’s correlation analysis was performed to explore the relationship between the donor and recipient’s psychological states concerning the MMPI-2, STAI, and CES-D. Specifically, we calculated the correlation coefficients between the biologically related and unrelated groups. *P*-values with an alpha level of 0.05 were considered significant, except that a Bonferroni-corrected significance level of *p* < 0.017 (0.05/3 tests) was applied to the comparisons of the psychological assessments between the recipients and donors in three different groups (whole, biologically related, and biologically unrelated group, respectively). All the data were analyzed using SAS software version 9.4 (SAS Institute Inc., Cary, NC, USA).

## Results

The socio-demographic characteristics of recipients and donors are presented in Table 1. The mean ages of the recipient and donor groups at the time of the evaluation were 46.98 ± 11.27 and 49.36 ± 11.31 years, respectively. Regarding gender, 64.15% of the total recipient sample identified themselves as male and 35.85% as female, while 41.51% of the entire donor sample identified themselves as male and 58.49% as female.

### Comparison of the psychological assessments between recipients and living donors

The comparison of psychological assessments between recipients and donors for the sub-scales of MMPI-2, STAI, and CES-D is given in Table 2 with a Bonferroni-corrected significance level of *p* < 0.017 (0.5/3 tests). When comparing the recipient and donor groups in the total sample, the former showed significantly higher scores for Hypochondriasis (*t* = − 4.49, *p* = .0001), Depression (*t* = − 3.36, *p* = .0015), Hysteria (*t* = − 3.30, *p* = 0.0018), and CES-D (*t* = − 3.93, *p* = .0003) than the latter. The biologically unrelated recipient group reported higher scores of Hypochondriasis (*t* = − 3.37, *p* = .003) and Depression (*t* = − 2.86, *p* = 0.0098) than the biologically unrelated donor group. Higher scores for Hypochondriasis (*t* = − 3.00, *p* = 0.0054) and CES-D (*t* = − 3.53, *p* = .0014) were found in the biologically related recipient group than the biologically related donor group.

**Table 2:** Comparison and correlations of the psychological assessments between kidney transplant recipients and donors (Mean (*SD*))

Total sample						Biologically related sample				Biologically unrelated sample			
Variables	Recipient (*n* = 53)	Donors (*n* = 53)	*t*	*P*	Spearman’s Correlation(*P*)	Recipient (*n* = 31)	Donors (*n* = 31)	*t*	*P*	Recipient (*n* = 22)	Donors (*n* = 22)	*t*	*P*
MMPI_L	49.89(8.09)	50.25(9.26)	0.02	0.98	0.03	47.74(6.93)	50.55(10.89)	1.23	0.23	52.91(8.78)	49.81(6.35)	−1.96	0.06
MMPI_F	43.32(6.47)	44.29(7.65)	0.70	0.49	0.22	43.94(7.16)	45.26(8.34)	0.75	0.46	42.45(5.40)	42.86(6.42)	0.14	0.89
MMPI_K	52.55(9.59)	52.92(11.22)	0.14	0.89	0.28†	51.13(10.26)	52.71(12.76)	0.67	0.51	54.55(8.39)	53.24(8.74)	−0.69	0.50
MMPI_Hs	51.57(8.66)	44.79(6.13)	−4.49	0.0001^*^	−0.06	50.97(8.20)	45.03(5.85)	−3.00	0.0054^*^	52.41(9.41)	44.43(6.67)	−3.37	0.003^*^
MMPI_D	53.30(11.15)	46.87(8.79)	−3.36	0.0015^*^	0.02	52.97(11.34)	46.90(9.78)	−2.16	0.04	53.77(11.12)	46.81(7.33)	−2.86	0.0098^*^
MMPI_Hy	51.74(8.80)	46.29(7.20)	−3.30	0.0018^*^	−0.10	50.52(7.84)	46.13(7.26)	−2.11	0.04	53.45(9.92)	46.52(7.27)	−2.57	0.02
MMPI_Pd	47.43(10.63)	44.69(8.23)	−1.43	0.16	−0.06	47.65(11.76)	44.77(9.29)	−0.98	0.34	47.14(9.06)	44.57(6.56)	−1.20	0.25
MMPI_Mf	50.06(9.65)	46.65(7.83)	−1.77	0.08	−0.07	49.61(10.62)	47.55(7.21)	−0.88	0.39	50.68(8.31)	45.33(8.69)	−1.78	0.09
MMPI_Pa	46.81(8.16)	45.75(8.83)	−0.68	0.50	−0.01	47.87(9.88)	46.45(9.91)	−0.54	0.59	45.32(4.64)	44.71(7.05)	−0.44	0.67
MMPI_Pt	46.06(7.65)	45.12(9.30)	−0.56	0.58	0.06	45.90(8.61)	45.68(8.19)	−0.10	0.92	46.27(6.23)	44.29(10.90)	−0.77	0.45
MMPI_Sc	43.77(8.08)	44.44(8.79)	0.40	0.69	0.08	43.77(9.45)	45.35(7.90)	0.75	0.46	43.77(5.84)	43.10(10.02)	−0.32	0.76
MMPI_Ma	45.79(8.84)	46.27(8.32)	0.39	0.70	0.40†	43.61(5.65)	46.26(9.04)	1.66	0.11	48.86(11.44)	46.29(7.34)	−0.91	0.37
MMPI_Si	46.43(9.98)	47.63(10.41)	0.50	0.63	0.19	47.45(10.31)	48.16(10.57)	0.28	0.78	45.00(9.55)	46.86(10.37)	0.44	0.67
STAI-S	39.91(10.45)	37.29(10.42)	−1.50	0.14	0.36†	39.16(10.88)	35.71(10.66)	−1.45	0.16	40.95(9.98)	39.62(9.84)	−0.48	0.64
STAI-T	39.81(10.32)	35.96(9.08)	−2.14	0.04	0.26	40.13(11.00)	35.32(9.25)	−1.93	0.06	39.36(9.51)	36.90(8.97)	−0.92	0.37
CES-D	13.49(8.24)	8.56(6.77)	−3.93	0.0003^*^	0.36†	13.61(9.45)	7.94(6.44)	−3.53	0.0014^*^	13.32(6.35)	9.48(7.30)	−1.88	0.08

*Note: MMPI-2* Minnesota Multiphasic Personality Inventory-2, *L* Lie, *F* Infrequency; *K* Defensiveness, *Hs* Hypochondriasis, *D* Depression, *Hy* Hysteria, *Pd* Psychopathic Deviate, *Mf* Masculinity-Femininity, *Pa* Paranoia, *Pt* Psychasthenia, *Sc* Schizophrenia, *Ma* Hypomania, *Si* Social Introversion, *STAI-S* Spielberger State-Trait Anxiety Inventory-State, *STAI-T* Spielberger State-Trait Anxiety Inventory-Trait, *CES-D* The Center for Epidemiologic Studies Depression Scale

^*^p < 0.017 (Bonferroni-corrected significance level; 0.5/3 tests)

†*p* < 0.05 (Spearman’s correlation analyses)

### Correlations of the psychological assessments between recipients and living donors

Table 2 contains the correlations between the MMPI-2, STAI-S, STAI-T, and CES-D sub-scores of recipients and donors. In MMPI-2, positive correlations between recipients and donors on the Hypomania (*r* = .40, *p* = .003) sub-scale were found. Significant positive correlations were seen in the STAI-S (*r* = 0.36, *p* = .009) and CES-D (*r* = 0.36, *p* = .008) scores. The correlations between the sub-scales of biologically related and unrelated recipient-donor pairs are given in the Supplementary Tables 1 and 2, respectively. As opposed to the biologically unrelated group, a positive correlation between the Hypomania (*r* = .47, *p* = .008) and CES-D (*r* = .42, *p* = .0202) sub-scores was observed in the biologically related pairs, while a significant correlation was observed between biologically unrelated recipients and donors in the STAI-T score (*r* = .52, *p* = .016). Furthermore, an association tendency was also found in the STAI-S score (*r* = .41, *p* = .063) that was not significant.

## Discussion

Comparison of the psychological characteristics of recipients and donors shows that the scores for the Hypochondriasis, Depression, and Hysteria sub-scales in the MMPI-2 were significantly higher in the recipient group. The Hypochondriasis, Depression and Hysteria sub-scales constitute the “neurotic triad” [34], i.e., high scores on all three scales are associated with an excessive concentration on somatic health status as well as frequent complaints of physical illnesses [35]. As with various chronic diseases, many physical and psychological stressors exist during the course of ESRD [36]. One prospective cohort study reported that kidney transplantation recipients’ anxiety and depression symptoms progressively increase during the wait for transplantation. Considering that the average waiting time for kidney transplantation in South Korea is 1592 days [37], It is obvious that patients suffer from depression, anxiety, and deterioration of quality of life with various stresses (e.g., uncertainty about life and death, social isolation, and economic problems) [38]. Despite dialysis therapy, transplantation recipients with ESRD present a high morbidity rate of cardiovascular diseases due to atherosclerosis and vascular calcification. The incidence of malignant tumors is also higher in recipients than the general population [12]. Even after transplantation, recipients are faced with persistent medical sequelae requiring strict medical surveillance and the maintenance of immunosuppression.

The total CES-D (depression) scores were also higher in the recipient group, reflecting the depressive state of the patients. This seems to be a result of the recipients’ cumulative hopelessness, uncertainty, and depression caused by the long waiting periods, as well as the lifestyle disruption due to chronic physical illness and hemodialysis [38, 39]. This finding suggests that clinician must consider the fact that the psychological difficulties are generally more severe in recipients than in donors. Although not examined in this study, further studies will be needed to determine whether more severe depression and elevated “neurotic triads” in recipients may affect long-term transplantation outcomes prospectively.

Interestingly, the correlations between the psychological assessments and STAI-S (state-anxiety) and CES-D (depression) scores of recipients and donors showed significant positive correlations between donors and recipients. This means that the more severe the recipient’s symptoms of depression and anxiety, the more severe the donor’s symptoms of depression and anxiety. Psychological difficulties like depression have a characteristic known as “emotional contagion” [40, 41]. This has been conceptualized under the term “depression contagion” and interpreted through the interactional theory of depression [42–44]. Depressed moods easily spread through intimate contacts among friends and families over long periods of time [45, 46]. Similarly, anxiety symptoms are emotionally contagious [47]. According to this theory, recipients who have suffered from chronic illness could share their negative emotions such as depression and anxiety during the long disease course with emotionally related donors. Therefore, the clinician should carefully evaluate the mood state of the recipient, who may be vulnerable to depression and anxiety, and when the recipient has symptoms such as depression and anxiety, the donor’s mood state should be evaluated in detail. Clinicians should also be aware of donor–recipient relationships and stressful situations in the family environment.

Additionally, when we compared biologically related and unrelated recipients and donors, some different results were found (Supplementary Tables 1 and 2). First, the depression (CES-D) score was positively correlated in biologically related recipients and donors. However, in the biologically unrelated but emotionally related recipients and donors, the higher the recipients’ anxiety (STAI-S, STAI-T) scores, the higher the donors’ anxiety scores. In South Korea, of 1260 people who received living donor kidney transplantation, 431 (34.20%) donations were by spouses, who are biologically unrelated but emotionally related [3]. Though we were unable to distinguish specific relationships between donors and recipients in this study, these results show that most of the biologically unrelated but emotionally related donors are in marital relationships. In this case, although several strategies have been used to control graft rejection in recent years, there is an anxiety about HLA (human leukocyte antigen) incompatibility in biologically unrelated pairs. It also has clinical significance, as clinicians have to assess the anxiety status of recipients and donors carefully, especially in an emotionally related relationship, and explore the reason for the anxiety in order to improve the prognosis of transplantation.

This study has several limitations. First, the sample size was relatively insufficient, as we only collected our sample at a single hospital. We should be careful in generalizing the results to living kidney transplantation donors and recipients with different characteristics. We were also limited in the availability of basic information such as education and occupation. In addition, given the cross-sectional nature of this study, it was impossible to identify the psychological and medical prognosis of donors and recipients. Further studies focusing on the effects of psychological prognoses (e.g., depression, anxiety, donor-recipient relationship, and emotional contagion) and medical prognoses (e.g., infection, rejection, and mortality) can aid in understanding recipients and donors’ psychological characteristics and their associations in detail. Lack of information about the stress factors affecting patients’ scores on various psychological scales, including family dynamics and socioeconomic status, also limits this study. Multiple testing is another limitation. When we compared the psychological assessments between recipients and donors, we set statistical significance at *p* < .017 by applying the Bonferroni correction. However, the rest of the analysis was not conducted with corrected statistical significance because it was intended to identify potential differences for each item. Type 1 error might have been increased thereby. Finally, although kidney transplantation is divided into biologically related and unrelated pairs, additional details on the types of relationships between donors and recipients were not assessed. Thus, we assumed the types of relationships with reference to the national statistics for transplantation.

## Conclusion

This study found that transplantation recipients suffered from a higher level of depression than donors before living kidney transplantation. The somatic concerns of the recipient group were also higher than in the donor group. Both donors and recipients can experience depression and anxiety. Specifically, positive correlations between recipients and donors on the Hypomania of MMPI-2, STAI-S, and CES-D scores. These results confirm that clinicians must pay greater attention to mood symptoms, including anxiety and depression, not only in recipients but also in donors because of emotional contagion.

## Supplementary information

**Additional file 1: Supplementary Table 1.** Correlations of the psychological assessments between biologically related recipients and donors (*n* = 62). **Supplementary Table 2.** Correlations of the psychological assessments between biologically unrelated recipients and donors (*n* = 44)

## Data Availability

The datasets analyzed during the current study are available from the corresponding author upon reasonable request.

## Abbreviations

ESRDf: End Stage Renal Disease
IRODaT: International Registry in Organ Donation and Transplantation
MMPI-2: Minnesota Multiphasic Personality Inventory-2
Hs: Hypochondriasis
D: Depression
Hy: Hysteria
Pd: Psychopathic Deviate
Mf: Masculinity-Femininity
Pa: Paranoia
Pt: Psychasthenia
Sc: Schizophrenia
Ma: Hypomania
Si: Social Introversion
L: Lie
F: Infrequency
K: Defensiveness
STAI: State-Trait Anxiety Inventory
STAI-T: State-Trait Anxiety Inventory Trait Anxiety Subscale
STAI-S: State-Trait Anxiety Inventory State Anxiety Subscale
CES-D: The Center for Epidemiologic Studies Depression Scale
ELPAT: Ethical, Legal and Psychosocial Aspects of Organ Transplantation
HLA: Human Leukocyte Antigen
