# Supplementary material for: Psychological characteristics and associations between living kidney transplantation recipients and biologically related or unrelated donors
Source: BMC Nephrol. 2020 Aug 20;21:355. doi: 10.1186/s12882-020-02017-y (PMC7441633; doi:10.1186/s12882-020-02017-y)
Supplement: Supplementary file 1 — Additional file 1: Supplementary Table 1. Correlations of the psychological assessments between biologically related recipients and donors (n = 62). Supplementary Table 2. Correlations of the psychological assessments between biologically unrelated recipients and donors (n = 44) [file 12882_2020_2017_MOESM1_ESM.pdf]

Supplementary Tables

Psychological characteristics and associations between living kidney transplantation recipients and biologically related or unrelated donors

Yujin Lee, M.D.[1†], Hyewon Park, M.S.[2†], Hee-Jung Jee, Ph.D.[3], Heon-Jeong Lee, M.D., Ph.D.[4], Jun Gyo Gwon, M.D., Ph.D.[5], Hyeonjin Min, M.D.[6], Cheol Woong Jung M.D., Ph.D.[5], Myung-Gyu Kim M.D., Ph.D.[6], Chul-Hyun Cho, M.D., Ph.D.[7,8*]

[1]Department of Psychiatry, Seoul Metropolitan Eunpyeong Hospital, Seoul, South Korea

[2]Department of Psychiatry, Samsung Biomedical Research Institute, Seoul, South Korea

[3]Department of Biostatistics, Korea University College of Medicine, Seoul, South Korea

[4]Department of Psychiatry, Korea University College of Medicine, Seoul, South Korea

[5]Department of Surgery, Korea University College of Medicine, Seoul, South Korea

[6]Division of Nephrology, Department of Internal Medicine, Korea University College of Medicine, Seoul, South Korea

[7]Department of Psychiatry, Chungnam National University Sejong Hospital, Sejong, South Korea

[8]Department of Psychiatry, College of Medicine, Chungnam National University, Daejeon, South Korea

† The first two authors contributed equally to this work.

*Corresponding Author

Chul-Hyun Cho, M.D., Ph.D.

Department of Psychiatry, College of Medicine, Chungnam National University, Daejeon, South Korea

82 Munhwa-ro, Jung-gu, Daejeon 35015, Republic of Korea

Tel: 044-995-4775. E-mail: david0203@gmail.com

Supplementary Table 1 Correlations of the psychological assessments between biologically related recipients and donors (n = 62)

Recipient's psychological dimensions	Donor's psychological dimensions															
	L	F	K	Hs	D	Hy	Pd	Mf	Pa	Pt	Sc	Ma	Si	STAI-S	STAI-T	CES-D
L	−0.03															
F		0.20														
K			0.37*													
Hs				−0.09												
D					−0.09											
Hy						−0.12										
Pd							−0.11									
Mf								−0.09								
Pa									−0.004							
Pt										−0.04						
Sc											0.06					
Ma												0.47*				
Si													0.19			
STAI-S														0.30		
STAI-T															0.12	
CES-D																0.42*

*$p < 0.05$.

Note: MMPI-2 = Minnesota Multiphasic Personality Inventory-2; L = Lie; F = Infrequency; K = Defensiveness; Hs = Hypochondriasis; D = Depression; Hy = Hysteria; Pd = Psychopathic Deviate; Mf = Masculinity-Femininity; Pa = Paranoia; Pt = Psychasthenia; Sc = Schizophrenia; Ma = Hypomania; Si = Social Introversion; STAI-S; Spielberger State-Trait Anxiety Inventory-State; STAI-T; Spielberger State-Trait Anxiety Inventory-Trait; CES-D; The Center for Epidemiologic Studies Depression Scale.

Supplimentary Table 2 Correlations of the psychological assessments between biologically unrelated recipients and donors (n = 44)

Recipient's psychological dimensions	Donor's psychological dimensions															
	L	F	K	Hs	D	Hy	Pd	Mf	Pa	Pt	Sc	Ma	Si	STAI-S	STAI-T	CES-D
L	0.11															
F		0.28														
K			0.11													
Hs				−0.01												
D					0.17											
Hy						−0.08										
Pd							0.09									
Mf								−0.01								
Pa									−0.04							
Pt										0.24						
Sc											0.22					
Ma												0.30				
Si													0.20			
STAI-S														0.41		
STAI-T															0.52*	
CES-D																0.33

*$p < 0.05$.

Note: MMPI-2 = Minnesota Multiphasic Personality Inventory-2; L = Lie; F = Infrequency; K = Defensiveness; Hs = Hypochondriasis; D = Depression; Hy = Hysteria; Pd = Psychopathic Deviate; Mf = Masculinity-Femininity; Pa = Paranoia; Pt = Psychasthenia; Sc = Schizophrenia; Ma = Hypomania; Si = Social Introversion; STAI-S; Spielberger State-Trait Anxiety Inventory-State; STAI-T; Spielberger State-Trait Anxiety Inventory-Trait; CES-D; The Center for Epidemiologic Studies Depression Scale.
